# The impact of neonatal intensive care unit antibiotics on gut bacterial microbiota of preterm infants: a systematic review

**DOI:** 10.3389/frmbi.2023.1180565

**Published:** 2023-07-28

**Authors:** Martin M. Mulinge, Sylviah S. Mwanza, Hellen M. Kabahweza, Dalton C. Wamalwa, Ruth W. Nduati

**Affiliations:** ^1^ Department of Biochemistry, School of Medicine, University of Nairobi, Nairobi, Kenya; ^2^ Kenya AIDS Vaccine Initiative - Institute of Clinical Research, University of Nairobi, Nairobi, Kenya; ^3^ Behavioural and Chemical Ecology Department, International Centre of Insect Physiology and Ecology (ICIPE), Nairobi, Kenya; ^4^ Department of Pediatric Hematology & Oncology, Joint Clinical Research Centre, Kampala, Uganda; ^5^ Department of Paediatrics and Child Health, University of Nairobi, Nairobi, Kenya

**Keywords:** antibiotics, dysbiosis, gut microbiota, NICU, preterm infant, 16S rRNA sequencing

## Abstract

Preterm infants encounter an unnatural beginning to life, with housing in neonatal intensive care units (NICUs) where they are exposed to antibiotics. Although the effectiveness of antibiotics in infection control is well established, the short- and long-term unintended effects on the microbiota of preterm infants receiving antibiotic treatment are yet to be quantified. Our aim was to investigate the unintended consequences of NICU antibiotics on preterm infants’ gut microbiota. We searched three electronic databases—Embase, PubMed, and Scopus—for records from 2010 to October 2022. Eligibility criteria included intervention and observational studies that collected stool samples and analyzed microbiota data on the effect of antibiotics on the gut microbiota of preterm infants using 16S rRNA sequencing. The Preferred Reporting Items for Systematic Reviews and Meta-Analyses (PRISMA) guidelines were followed, and the quality of the studies was judged using the Cochrane Collaboration Tool for assessing risk of bias (RoB2) for clinical trials, while non-randomized studies were assessed using the Newcastle–Ottawa Scale (NOS). The initial searches yielded 7,605 papers, of which 21 were included in the review. The selected studies examined 3,669 stool samples that were collected longitudinally from 878 preterm infants in seven different countries. Preterm infants exposed to antibiotics had a reduced bacterial diversity, an increased relative abundance of pathogenic bacteria such as Enterobacteriaceae, and a decrease or absence of symbiotic bacteria such as *Bifidobacterium* spp., which have been shown to assist in immunity development. Antibiotic discontinuation restored diversity, with variances linked to the antibiotic spectrum and treatment duration in some but not all cases. Breastfeeding confounded the association between antibiotic use and dysbiosis. Intriguingly, the reduction of γ-aminobutyric acid (GABA), a crucial neurotransmitter for early brain development, was linked to the depletion of *Veillonella* spp. Despite the apparent benefits of using antibiotics on preterm infants, we conclude that they should be used only when absolutely necessary and for a short period of time. Mothers’ milk is recommended to hasten the restoration of disrupted microbiota.

## Introduction

Advances in next-generation sequencing, as well as increased funding for microbiome projects, have enhanced our knowledge of how the microbiome influences human health and disease. The advent of metagenomics, a culture-independent technique that utilizes 16S rRNA sequencing, has facilitated the characterization of the gut microbiota that are currently not culturable (Turroni et al., 2020). This has called into question the long-held belief that the uterus is a sterile environment, after microbes were discovered in the placenta, amniotic fluid, fetal membrane, umbilical cord blood, and meconium, as reviewed in (Tamburini et al., 2016). The assembly of microbial communities within the gastrointestinal tract during early life plays a critical role in immune, endocrine, and metabolic functions, especially in the first 1,000 days of life (Robertson et al., 2019; Aires, 2021). It is extremely important for preterm infants, who are developmentally immature and are forced to develop outside of the womb, where they are exposed to factors such as antibiotics, parenteral nutrition, and stress, all of which could interfere with the development of their early-life microbiota (Robertson et al., 2019). Other factors that have an influence on gut microbiota include maternal diet, which has an influence on breastmilk composition, probiotics, postnatal age, and mode of delivery (Paganini et al., 2019; Korpela, 2021; Li et al., 2022). Long-term effects of disturbed human–microbiota interactions include the occurrence of allergic diseases (Keeney et al., 2014; Riiser, 2015), obesity (Rautava, 2021), and neurodevelopmental conditions (Lacorte et al., 2019; Patangia et al., 2022). Thus, preterm microbiota research is critical and understanding the effect of antibiotics on microbiota colonization patterns can help pediatricians and neonatal intensive care unit (NICU) caregivers develop interventions to promote infant health in this vulnerable population.

The majority of preterm infants are exposed to antibiotics while in the NICU. Despite sterile cultures and a low incidence of culture-proven bacterial sepsis, nearly all extremely low birth weight (ELBW) infants admitted to an NICU are given an empirical antibiotic treatment in the first postnatal days. A study of 6,956 very low birth weight (VLBW) infants by the National Institute of Child Health and Human Development National Research Network found that 56% of all infants received at least one course of antibiotic treatment, even though proven sepsis was diagnosed in only 21% of all infants (Stoll et al., 2002). While antibiotics reduce mortality and morbidity rates, they also disrupt gut microbiota development, and infants who receive early antibiotics have more cases of necrotizing enterocolitis (NEC), sepsis, or death than those who are not exposed to antibiotics (Greenwood et al., 2014). Such disturbances are characterized by low diversity and richness, with high interindividual variability compared with term infants (Wang et al., 2009; Dobbler et al., 2017); delayed *Bifidobacterium* spp. colonization (Zwittink et al., 2018); and increased presence of antibiotic resistance genes in members of *Enterococcus* spp.*, Staphylococcus* spp.*, Klebsiella* spp.*, Acinetobacter* spp.*, Pseudomonas aeruginosa*, and other Enterobacteriaceae (ESKAPE), which are also the most frequent causes of nosocomial infections in preterm neonates (Vangay et al., 2015; Gasparrini et al., 2019; Chen et al., 2020; Reyman et al., 2022). Notably, the gut is the main site of interaction between the host immune system and commensal or pathogenic microbes in early life. The levels of keystone bacteria such as *Bifidobacterium longum* are very critical due to their role in maturation of dendritic cells in Peyer’s patches and the development of T cells in the thymus (Dong et al., 2010; Alsharairi, 2023), and specific microbial signals are critical for proper education of regulatory T cells (Hansen et al., 2012).

Additionally, preterm infants with VLBW, i.e., with a birth weight of 1,500 g, experience feeding intolerance due to gastrointestinal tract underdevelopment, which impairs motility and nutrient absorption, resulting in abdominal distension, vomiting, and gastric retention (Unger et al., 2015). These factors threaten the development of the commensal gut microbiota and appear to be ameliorated by breast milk, with preterm infants fed with their mother’s milk developing fewer cases of sepsis and NEC, and incurring fewer NICU costs (Patel et al., 2013). However, maternal milk is limited for preterm infants because the production of maternal colostrum is limited after birth or intestinal immaturity hinders full enteral feedings.

While some systematic reviews have reviewed and synthesized data on the effect of antibiotic therapy on neonatal gut microbiota, reporting reductions in abundances of commensal anaerobic bacteria and decreased microbial diversity due to antibiotic therapy, with worse outcomes associated with long-term exposure (>7 day treatment) (Fjalstad et al., 2018; Zimmermann and Curtis, 2019a; Aguilar-Lopez et al., 2021; Morreale et al., 2023), to the best of our knowledge there are no systematic reviews that focus exclusively on the impact of specific antibiotics administered in the NICU on preterm gut microbiota. Our focus on antibiotic-induced microbiota changes in preterm neonates using culture-independent 16S rRNA sequencing was unique to this study. We anticipated that such a study would reveal new insights and inform the care of this highly vulnerable population, which was estimated to be 10.6% globally in 2014 (Chawanpaiboon et al., 2019). While antibiotic therapy is characterized by intended effects, there are unintended effects. The unintended antibiotic effects range from minor perturbations or “dysbiosis” in gut microbiota that may recover to the basal state to potentially life-threatening adverse effects. In order to avoid unnecessary treatments that could have long-term effects, it is crucial to use antibiotics to prevent infection in preterm infants based on evidence. Thus, the primary objective of this systematic review was to examine the unintended consequences of antibiotic use on preterm gut microbiota. For clarity, “microbiota” refers to microbial taxa within a certain ecosystem, whereas “microbiome” refers to microbial taxa and their genomes.

## Methods

This systematic review was conducted in accordance with the Preferred Reporting Items for Systematic Reviews and Meta-analyses (PRISMA) guidelines (Page et al., 2021). Since this was a review of published studies, ethical approval was not required.

### Data sources and search strategy

A systematic search was conducted in three databases: Embase, PubMed, and Scopus. The search terms used are listed in **Supplementary Table 1**; in brief, we included terms corresponding to three concepts: preterm infants, microbiome, and antibiotics. Boolean operators “AND” between concepts and “OR” within the concepts ensured the search was as comprehensive as possible. We conducted the search and limited the selection to original articles published in English, excluding studies from gray literature. The search was limited to studies published from January 2010 to 30 November 2022, when culture-independent 16S rRNA sequencing was widely used for microbiome characterization.

### Eligibility criteria

As the eligibility criteria, we developed a “patient, intervention, comparators, outcome, and study design” approach, guided by the following research question: how does antibiotic therapy [intervention] affect the gut microbiome [outcome] in preterm infants [population] when compared with no antibiotics [control]? Population: studies were eligible if they reported gut microbiota of preterm infants. Intervention: antibiotics administered within an NICU environment. Comparators: preterm infants with no exposure to antibiotics in similar settings. Outcome: gut bacterial microbiome identified using 16S rRNA sequencing with α- and/or β-diversity metrics reported. Study design: observational and intervention studies. Exclusion: articles were excluded if they were of an inappropriate study type (animal studies, *in vitro* studies, conference abstracts, reviews, and meta-analyses), did not associate gut microbiome composition and antibiotic therapy, or were published before 2010. The literature selection was conducted for eligibility by two authors (MM and SM), by first removing all clearly irrelevant articles, followed by abstract screening and finally full-text screening aided by rayyan (http://rayyan.qcri.org), a free web and mobile app that helps expedite the initial screening of abstracts and titles using a process of semi-automation while incorporating a high level of usability (Ouzzani et al., 2016). This tool has been used to conduct systematic reviews for similar studies (Leumi et al., 2018; Delbeke et al., 2020; Muka et al., 2020; Tadesse et al., 2020; Attaye et al., 2022). Discrepancies regarding study inclusion between the two authors were resolved through a discussion with HK.

### Data extraction

The data extracted from each article included author, year of publication, country, study design, study size (n), antibiotics, antibiotic-exposed group (n), controls (n), stool samples sequenced, follow-up time, length of treatment, 16S rRNA target, sequencing platform, α-diversity, and microbial taxa. MM, SM, and RN independently extracted data into a Microsoft Excel template. The demographic, antibiotic, and taxonomic data from the 21 studies are all detailed in **Supplementary Table 2**. In the situation of disagreements, a consensus was reached after a discussion among the three authors.

### Quality assessment

The study quality was evaluated independently by MM, SM, and HK. The eligible clinical trials were assessed using the Cochrane Collaboration Tool for assessing risk of bias (RoB2) (Sterne et al., 2019), while non-randomized studies were assessed using the Newcastle–Ottawa Scale (NOS) (Wells et al., 2017). The RoB2 tool assesses potential biases in five domains: randomization, deviations from intended interventions, missing outcome data, measurement of the outcome, and selection of the reported results. The NOS measures four domains: participant selection, comparability, exposure, and outcome. The scoring is based on the number of stars, with cross-sectional studies receiving up to six stars and longitudinal studies receiving up to nine stars.

### Data analysis

The primary objective across all the studies was to detect changes in composition of the bacterial microbiome following antibiotic exposure, with a focus on three main outcomes: identification of taxonomic composition, the relative abundance of identified taxa, and change in species richness/diversity, as indicated in **Supplementary Table 2**. Kruskal–Wallis tests and rank-sum tests were used to identify statistically significant differences in continuous variables, including gestational age and birth weight. The Fisher’s exact test was used to identify statistically significant differences in categorical variables, including number of samples collected per preterm infant, 16S rRNA target, and study design.

## Results

### Eligible studies

**Figure 1:**
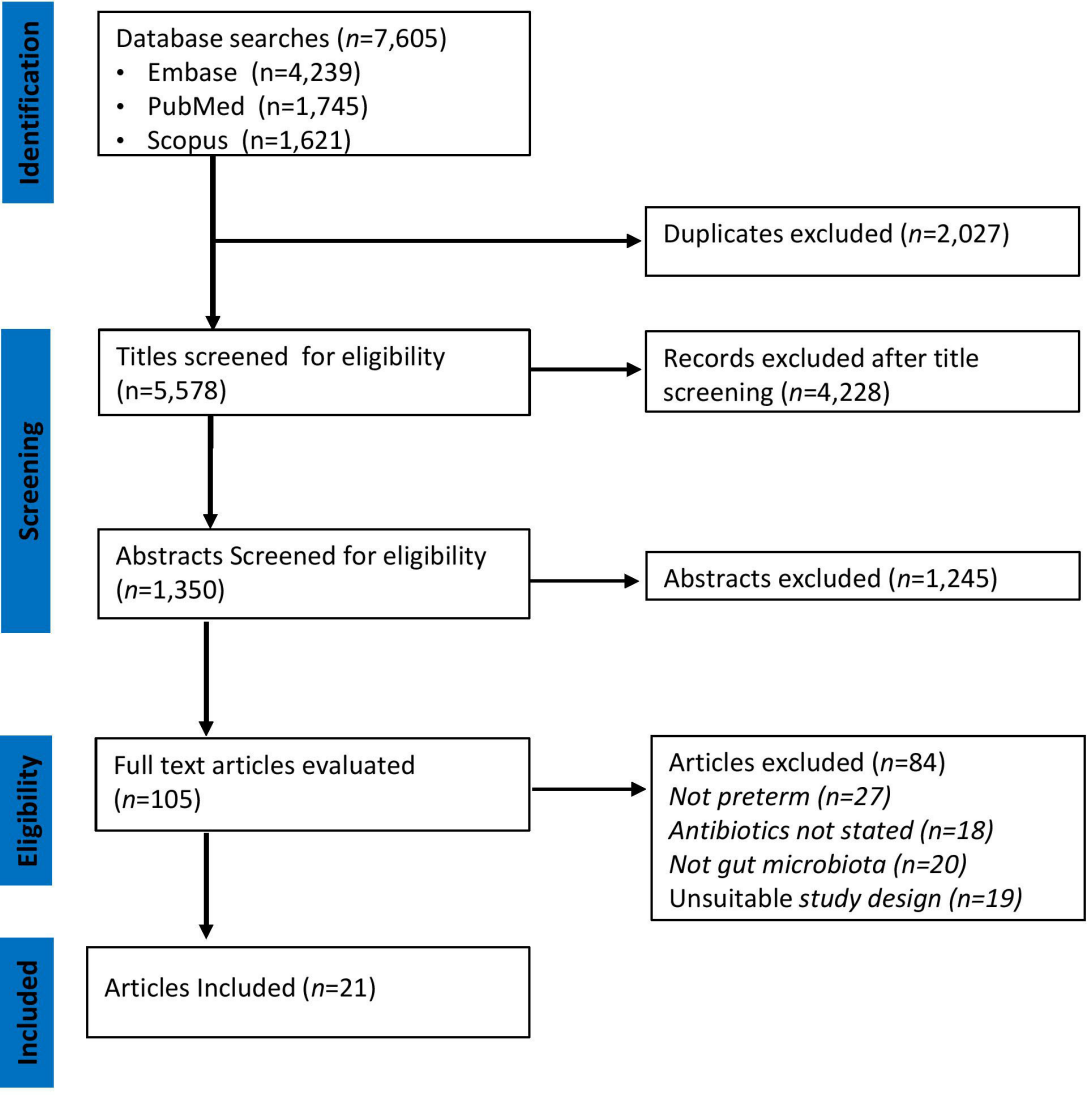
PRISMA Diagram of Study Selection. The relevant number of articles at each step is indicated.

A PRISMA flowchart illustrating the selection of studies is shown in **Figure 1**. The initial search in the three databases generated 7,605 records. A total of 2,027 publications were identified as duplicates using rayyan and were resolved. After removing duplicates, some 5,578 records remained. A further 5,473 were excluded after assessing titles and abstracts based on study type, missing data, or irrelevance. The full texts of the remaining 105 articles were downloaded and reviewed for eligibility by MM, SM, and HK. At this stage, 84 articles were excluded because they did not meet the inclusion criteria described in **Figure 1**. Finally, 21 articles [(two randomized controlled trials (Korpela et al., 2018; Russell et al., 2021) and 19 cohort studies (Barrett et al., 2013; Dardas et al., 2014; Drell et al., 2014; Greenwood et al., 2014; Arboleya et al., 2015; Gibson et al., 2016; Ravi et al., 2017; Zhu et al., 2017; Hourigan et al., 2018; Wandro et al., 2018; Zou et al., 2018; Zwittink et al., 2018; D’Agata et al., 2019; d’Haens et al., 2019; Liu et al., 2019; Jia et al., 2020; Lindberg et al., 2020; Lu et al., 2020; Zwittink et al., 2020)] were included were included as shown in **Table 1**.

**Table 1:** Summary of included studies.

	Author, year, country	Study design	Population	Antibiotic	Confounding	Sequencer, 16S rRNA	Alpha diversity	Taxonomy
1	Korpela et al., 2018, Norway	Clinical trial	PT infants = 45 Antibiotic = n.s. Control = n.s. Stool samples = 262	Gentamycin, ampicillin, vancomycin, cephalosporin	Feeding mode, Gestational age	Illumina, V1–V3	Antibiotic use decreased α-diversity	⇩ *Enterococcus* spp., ⇩ *Bifidobacterium* spp. in response to gentamycin. ⇧ *Enterococcus* spp., ⇩ *Bifidobacterium* spp. in response to vancomycin.
2	Russell et al., 2021, Norway	Clinical trial	Total = 91 Antibiotic = 77 Control = 14 Stool samples = 656	Vancomycin, piperacillin	Feeding mode, Gestational age	Illumina, V3–V4	Shannon α-diversity was not significantly different between the treatment and controls.	Absence of *Veillonella* spp. in treatment group compared to pre-treatment levels, restored after treatment stoppage.
3	Zhu et al., 2017, China	Observational	PT infants = 36 Antibiotic = 24 Control = 12 Stool samples = 62	Penicillin–moxalactam (PM), piperacillin-tazobactam (PT)	–	Illumina, V3–V4	No significant difference in α-diversity between the treatment and control groups at either time point.	Treatment groups had ⇩ diversity and ⇧ *Streptococcus* spp., and ⇧ *Pseudomonas* spp. when compared to controls. PT treatment caused ⇧ *Enterococcus* spp., and ⇩ *Klebsiella* spp., ⇩ *Clostridium* spp., PM treatment ⇧ *Escherichia* spp., and ⇩ *Shigella* spp. PT group had diversity compared to PM group
4	Zou et al., 2018, China	Observational	PT infants = 28 Antibiotic = n.s. Control = n.s. Stool samples = 48	Cefazolin, cefuroxime	Antibiotic resistance, Feeding mode	Illumina, V3–V4	Shannon index showed no significant difference between the two groups on day 7 and day 14	⇩ Bacteroidetes, ⇩ *Bifidobacterium* spp., ⇧ *Staphylococcus* spp., ⇧ *Streptococcus* spp., ⇧ *Serratia* spp., ⇧ *Parabacteroides* spp. in the treatment group.
5	Liu et al., 2019, China	Observational	PT infants = 24 Antibiotic = 7 Control = 17 Stool samples = 192	Cefotaxime, piperacillin-tazobactam, metronidazole	NEC, LOS	Illumina, V3–V4	Shannon α-diversity decreased significantly from the early post-partum to early disease stage	⇧ *Bacillus* spp., ⇧ Solibacillus spp., before onset of NEC and LOS. ⇧ *Enterococcus* spp., ⇧ *Streptococcus* spp. and ⇧ *Peptoclostridium* spp. during NEC progression ⇧ *Klebsiella* spp. during LOS progression
6	Jia et al., 2020, China	Observational	PT infants = 91 Antibiotic = n.s. Control = n.s. Stool samples = 558	Clarithromycin	Infant sex, Birth weight, Feeding mode, Birth mode	Illumina, V3–V4	Shannon α-diversity was not associated with antibiotic use.	⇩ *Lactobacillus* spp. and ⇩ *Enterococcus* spp. after 7–14 days and >15 day antibiotic use compared to <7 day of use. ⇩ *Bifidobacterium* spp. after >15 days of antibiotic use
7	Lu et al., 2020, China	Observational	PT infants = 16 Antibiotic = 6 Control = 10 Stool samples = 47	*Piperacillin–tazobactam (90 mg/kg), *Cefuroxime (50 mg/kg), *Piperacillin–sulbactam (75 mg/kg), *Cefoperazone–sulbactam (50 mg/kg)	–	Illumina, V4	Shannon α-diversity was significantly reduced in the antibiotic treated infants on day 7.	⇩ Bacteroidetes, ; ⇧ *Enterococcus* spp. in the treatment group. Diversity was restored spontaneously after discontinuation of antibiotic treatment
8	Drell et al., 2014, Estonia	Observational	PT infants = 50 Antibiotic = n.s Control = n.s Stool samples = 118	Ampicillin, penicillin, gentamicin	Birth weight, Feeding type, LOS, Chorioamnionitis	Illumina, V3–V4	Shannon α-diversity index increased	⇧ *Staphylococcus* spp., ⇧ Enterobacteriaceae, ⇩ *Bifidobacterium* spp., and ⇩ *Lactobacillus* spp.
9	Barrett et al., 2013, Ireland	Observational	PT infants = 10 Antibiotic = 9 Control = 1 Stool samples = 20	Benzyl penicillin, gentamicin, teicoplanin, cefotaxime	Birth mode, Gestation age, Feeding mode	Roche, V4	α-diversity was not different in the different treatment groups.	⇧ Enterobacteriaceae, ⇩ *Bifidobacterium* spp., and ⇩ *Lactobacillus* spp. with antibiotic use ⇧ *Staphylococcus* spp. in infants with no antibiotic treatment.
10	Zwittink et al., 2018, Netherlands	Observational	PT infants = 15 Antibiotic = 6 Control = 9 Stool samples = 95	Amoxicillin, ceftazidime	Post-natal age, Feeding mode	Roche, V3–V5	Chao1 index showed that short and long antibiotic treatment did not affect community diversity.	⇩ *Bifidobacterium* spp. after short and long antibiotic treatment. Antibiotic treatment ⇩ Enterobacteriaceae, ⇧ *Enterococcus* spp. up to 2-weeks after treatment.
11	d’Haens et al., 2019, Netherlands	Observational	PT infants = 19 Antibiotic = 14 Control = 5 Stool samples = 38	*Vancomycin (10 mg/kg)	Breastfeeding, Birth weight, LOS	Illumina, V3–V4	Chao1 α-diversity index showed no difference in community richness	⇧ *Staphylococcus* spp., ⇩ *Bifidobacterium* spp., ⇩ *Pseudomonas* spp., and ⇩ Commonadaceae observed shortly after treatment.
12	Zwittink et al., 2020, Netherlands	Observational	PT infants = 63 Antibiotic = 35 Control = 28 Stool samples = 263	*Amoxicillin (100 mg/kg/day) *Ceftazidime (100 mg/kg/day)	Gestational age, Birth mode, Infant sex, Feeding mode	Illumina, V3–V4	α-diversity was not consistently affected by antibiotic treatment.	⇧ *Enterococcus* spp., ⇧ *Clostridium* spp., ⇧ *Staphylococcus* spp., ⇧ *Escherichia* spp., *Shigella* spp., ⇧ Bacteroidetes, ⇧ Enterobacteriaceae in PT Infants receiving long-term antibiotic treatment. ⇩ *Bifidobacterium* spp. and ⇩ *Streptococcus* spp. was observed in both short- and long term treatment.
13	Arboleya et al., 2015, Spain	Observational	PT infants = 40 Antibiotic = 27 Control = 13 Stool samples = 160	Ampicillin, gentamicin, vancomycin, gentamycin, clindamycin, teichomycin	Birth mode, Birth weight	Ion Torrent, V3–V4	At 30 days of age, α-diversity was different between antibiotic treatment group and controls.	⇧ Commonadaceae, ⇧ Staphylococcaceae, ⇧ Bifidobacteriaceae, ⇧ Streptococcaceae, ⇧ Actinobacteria, ⇧ Lactobacillales and ⇩ Enterobacteriaceae at 30 days of age in infants NOT exposed to antibiotics.
14	Greenwood et al., 2014, USA	Observational	PT infants = 74 Antibiotic = 61 Control = 13 Stool samples = 239	Ampicillin, gentamicin	NEC, LOS	Roche, V3–V5	α-diversity did not differ by antibiotic exposure at week 1. In weeks 2 and 3, α-diversity was significantly different across treatment groups.	⇧ *Staphylococcus* spp. after 1-day of treatment. ⇧ Enterobacteriaceae and ⇩ bacterial diversity after 5–7 days of empiric antibiotic treatment. Infants receiving early antibiotics experienced more cases of NEC, sepsis, or death than controls.
15	Dardas et al., 2014, USA	Observational	PT infants = 27 Antibiotic = n.s. Control = n.s. Stool samples = 54	Ampicillin, gentamicin, vancomycin, cefazolin, cefotaxime, erythromycin	Feeding mode	Roche, V1–V3	A lower α-diversity for longer treatment groups. No difference after 30 days of life.	⇧ Bacteroidetes regardless of the duration of antibiotics. ⇧ Actinobacteria, ⇧ Proteobacteria, ⇧ Firmicutes had a relative rise at day 30 after 2 days of exposure.
16	Gibson et al., 2016, USA	Observational	PT infants = 84 Antibiotic = 51 Control = 33 Stool samples = 401	Ampicillin, gentamicin, meropenem, cefotaxime, ticarcillin–clavulanate, vancomycin	Postmenstrual age, Feeding mode, Antibiotic resistance	Illumina, V4	Antibiotic exposure in PT infants was associated with significantly reduced species richness	Meropenem, cefotaxime and ticarcillin–clavulanate were associated with ⇩ species diversity. Gentamicin was NOT associated with diversity. Enrichment of antibiotic Resistance (AR) genes.
17	Ravi et al., 2017, USA	Observational	PT infants = 62 Antibiotic = 62 Control = 39 Stool samples = 160	Gentamicin, vancomycin, ampicillin	NEC, Birth weight, Antibiotic resistance, Hospital location	Illumina, V3–V4	No major differences in the α-diversity between the NEC-positive and NEC-negative infants.	⇧ Enterobacteriaceae with response to antibiotic use. Antibiotic usage led to selection of antibiotic-resistant bacteria.
18	Wandro et al., 2018, USA	Observational	PT infants = 32 Antibiotic = n.s. Control = n.s. Stool samples = 77	Ampicillin, cefotaxime, cefazolin, gentamicin, oxacillin, piperacillin, vancomycin	Feeding mode, Birth mode, NEC, LOS	Illumina, V3–V4	Antibiotic use was not associated with α-diversity.	⇧ Enterobacteriaceae, ⇧ *Enterococcus* spp., ⇧ *Staphylococcus* spp., Lack of *Bifidobacterium* spp.
19	Hourigan et al., 2018, USA	Observational	PT infants = 32 Antibiotic = 14 Control = 18 Stool samples = 64	Ampicillin, gentamicin, 1st–3rd generation cephalosporin	Hospital environment, Feeding mode, Antibiotic resistance	Illumina, V3–V4	No differences were observed in α diversity between the samples from the new and old NICU	No differences between *Bifidobacterium* spp., *Lactobacillus* spp., Enterobacteriaceae, Staphylococcaceae, and *Ureaplasma* spp. for the two NICU observations. More antimicrobial resistance genes in the new NICU.
20	D’Agata et al., 2019, USA	Observational	PT infants = 29 Antibiotic = n.s. Control = n.s. Stool samples = 116	Antibiotic used but not specified (coded binary)	Neonatal stress	Illumina, V4	Antibiotic use was not associated with α-diversity.	⇩ *Proteus* spp. and ⇧ *Citrobacter* spp. and ⇧ *Clostridium perfringens* was associated with antibiotic use. Males had lower *Proteus* spp. than females. ⇧ *Proteus* spp., and ⇧ *Veillonella* spp. in stressed PT infants
21	Lindberg et al., 2020, USA	Observational	PT infants = 10 Antibiotic = 5 Control = 5 Stool samples = 29	Ampicillin, gentamicin, vancomycin, 3rd generation cephalosporin, meropenem	NEC, Feeding mode	Illumina, V4	Cumulative antibiotic exposure was associated with a diversity of 0.47 OTUs for each extra day. Antibiotic exposure not associated with α-diversity.	⇧ Proteobacteria among NEC infants compared to non-NEC infants who had ⇧ Firmicutes

**⇧** Increase; **⇩** decrease; * indicates the antibiotic dosage; LOS, late-onset sepsis; n.s., not stated; NEC, necrotizing enterocolitis; NICU, neonatal intensive care unit; OTU, operational taxonomic units; PT, preterm; VLBW, very low birth weight.

### Quality assessment

We assessed the methodological quality and risk of bias in the 21 included studies using RoB2 and NOS as appropriate. All the studies included clearly defined research questions and specific objectives. One of the two intervention studies was of high quality (Russell et al., 2021), while the other study lacked clear randomized assignment of participants, did not clearly define concealment methodologies, and was ranked as being of moderate quality (Korpela et al., 2018). The quality of evidence for the 19 observational studies varied from very low to high (**Supplementary Table 3**). The key explanation for the observational studies’ lower quality was a lack of enough detail about potential baseline differences between groups or confounding variables.

### Study characteristics

The demographic, antibiotic, and taxonomic data of the included studies are summarized in **Supplementary Table 2**. The studies were conducted in the following countries: China (Zhu et al., 2017; Zou et al., 2018; Liu et al., 2019; Jia et al., 2020; Lu et al., 2020), Estonia (Drell et al., 2014), Ireland (Barrett et al., 2013), the Netherlands (Zwittink et al., 2018; d’Haens et al., 2019; Zwittink et al., 2020), Norway (Korpela et al., 2018; Russell et al., 2021), Spain (Arboleya et al., 2015), and the USA (Dardas et al., 2014; Greenwood et al., 2014; Gibson et al., 2016; Ravi et al., 2017; Hourigan et al., 2018; Wandro et al., 2018; D’Agata et al., 2019; Lindberg et al., 2020). The 21 studies included 878 preterm infants and 3,669 stool samples. The majority of the preterm infants were very preterm; the lowest mean gestational age was 25 weeks (Gibson et al., 2016) and the highest mean gestational age was 35.2 weeks (Lu et al., 2020). The lowest mean birth weight was 680g (Lindberg et al., 2020) and the highest mean birth weight was 2,871g.

### Next-generation sequencing platforms

The microbiome data for all 21 studies was generated using 16S rRNA gene sequencing (**Table 1**). In addition, two studies used shotgun metagenomic sequencing to profile antibiotic-resistant genes (Gibson et al., 2016; Ravi et al., 2017). The 16S rRNA gene contains nine hypervariable (V) regions (V1–V9) which have a high variability for identification of bacteria and archaea (Schloss and Westcott, 2011). Currently, the segments of V1–V3, V4, and V4–V5 regions are most commonly used because research has shown that each can provide genus-level sequence resolution, with the V1–V3 or V1–V4 regions providing more accurate estimates than others (Kim et al., 2011). In almost half (n = 10) of the studies, the V3–V4 region of the 16S rRNA was targeted, followed by the V4 region (n = 5); these were performed using the Illumina and Ion Torrent platforms. The studies that utilized the Roche 454 platform targeted other regions of the 16S rRNA and were performed between 2010 and 2018 (Barrett et al., 2013; Dardas et al., 2014; Greenwood et al., 2014; Zwittink et al., 2018). We sought to explore heterogeneity in the studies with respect to the variable region of the 16S rRNA gene targeted (V1–V3, V1–V4, V3–V4, V3–V5, and V4) and did not observe differences in α-diversity metrics with respect to the 16S rRNA target regions.

### Antibiotic-induced microbiota diversity changes

Different studies reported different taxa, including phylum, class, family, order, genus, and species. The α-diversity metrics reported by the included studies were inconsistent and varied from the mean or median value to a statement of whether it was significantly reduced or increased. The reported indexes included: Chao-1’s index, Shannon index, Simpson’s index, and Pielou’s evenness index. Antibiotic therapy was associated with a reduction in α-diversity in 17/21 studies, there were no changes in three studies (Hourigan et al., 2018; Wandro et al., 2018; Zwittink et al., 2020), and there was an increase in one study (Drell et al., 2014). Significant differences in β-diversity metrics were reported in 6/8 studies (Dardas et al., 2014; Ravi et al., 2017; Zou et al., 2018; d’Haens et al., 2019; Liu et al., 2019; Lu et al., 2020). Zou et al. examined the impact of cefazolin and cefuroxime over a 2-week interval and reported an unchanged α-diversity but differences in β-diversity in long-term exposed infants (>7 day) compared with short-term exposed infants (<7 days) (Zou et al., 2018). This means that although the individual diversity index did not change (i.e., wide species variety and abundance), there was a significant change in the types of species found. Similar results were reported by d’Haens et al., who focused exclusively on the effect of two prophylactic doses of vancomycin on gut microbiome (d’Haens et al., 2019).

### Decreased relative abundance of phylum Actinobacteria

The association between antibiotics and the relative abundance of genus *Bifidobacterium* spp. (a very important genus within the phylum Actinobacteria) was examined in 10 studies. In 9/10 studies, in antibiotic-treated infants the relative abundance of *Bifidobacterium* spp. decreased significantly (Barrett et al., 2013; Drell et al., 2014; Arboleya et al., 2015; Korpela et al., 2018; Zou et al., 2018; Zwittink et al., 2018; d’Haens et al., 2019; Jia et al., 2020; Zwittink et al., 2020). The most profound impact was the disappearance of *Bifidobacterium* spp. following ampicillin and gentamicin administration (Wandro et al., 2018). This dysbiosis did not last longer than 30 days in more than half of the studies, at which point the *Bifidobacterium* spp. population appeared to have recovered.

### Increased relative abundance of phylum Bacteroidetes

Two studies found an increase in the association between antibiotic exposure and the relative abundance of the phylum Bacteroidetes (Dardas et al., 2014; Zwittink et al., 2020). In their study, Dardas et al. (2014) reported an increase in Bacteroidetes after an exposure of ampicillin and gentamicin and this phylum remained co-dominant alongside Firmicutes for 30 days. Similarly, Zwittink et al. (2020) reported increases in relative abundance of phylum Bacteroidetes after >5 days of exposure to a combination of amoxicillin and ceftazidime. Notably, the increase in phylum Bacteroidetes was associated with a decrease in relative abundance of beneficial *Bifidobacterium* spp. Contradictory results were reported by Zou et al. (2018), who observed that Bacteroidetes and *Bifidobacterium* decreased significantly but there was an increase in communities comprising *Staphylococcus* spp.*, Streptococcus* spp.*, Serratia* spp., and *Parabacteroides* spp. in preterm infants treated with cefazolin and cefuroxime compared with controls. In rare instances, these microbes may cause disease if dysbiosis causes a bloom of pathogenic species at the expense of beneficial species. In the study by Lu et al., there was a significant reduction in *Bacteroides* and an increase in *Enterococcus* in the preterm infants treated with β-lactam antibiotics. The effect of the antibiotic exposure on bacterial diversity was restored spontaneously after discontinuation of the treatment (Lu et al., 2020).

### Increased relative abundance of phylum Proteobacteria

The association between antibiotics and the relative abundance of phylum Proteobacteria was examined in 7/21 studies. The relative abundance of Enterobacteriaceae (a family in phylum Proteobacteria) was increased following exposure to antibiotics utilized in all studies (Barrett et al., 2013; Drell et al., 2014; Greenwood et al., 2014; Arboleya et al., 2015; Ravi et al., 2017; Wandro et al., 2018; Zwittink et al., 2020). The relative abundance of *Serratia* spp. was increased after exposure to cefazolin and cefuroxime (Zou et al., 2018). The relative abundance of the genera *Escherichia* and *Shigella* increased in two studies in which preterm infants were exposed to penicillin-moxalactam (Zou et al., 2018). There was a change in relative abundance of *Klebsiella* spp.; however, the direction of association was not consistent. Liu et al. reported an increase in relative abundance in preterm infants exposed to cefotaxime, piperacillin–tazobactam, and/or metronidazole (Liu et al., 2019), while Zhu et al. (2017) found a significant reduction in the relative abundance of *Klebsiella* spp. in premature antibiotic-exposed infants compared with those exposed to penicillin–moxalactam and piperacillin–tazobactam. The relative abundance of genus *Proteus* spp. was significantly higher in NICU-stressed male preterm infants exposed to antibiotics (D’Agata et al., 2019).

### Bi-directional changes of phylum Firmicutes

There were conflicting findings between antibiotic use and the relative abundance of the phylum Firmicutes. Two authors reported a relative increase in Staphylococcaceae (Drell et al., 2014; Zwittink et al., 2020), while two authors reported a decrease or no change respectively (Arboleya et al., 2015; Hourigan et al., 2018). A similar trend was observed for the Streptococcaceae family, for which two authors reported an increase (Zhu et al., 2017; Liu et al., 2019) and two authors reported a decrease (Arboleya et al., 2015; Zwittink et al., 2020). The study by D’Agata et al. (2019) reported an increase in the relative abundance of *Veillonella* spp., which was completely absent in preterm infants exposed to vancomycin and piperacillin in the study of Russell et al. (2021). The consequence of this was the reduction of γ-aminobutyric acid (GABA), a critical neurotransmitter for early brain development (Russell et al., 2021). In 7/8 studies, an increase in relative abundance of *Enterococcus* spp. was reported (Zhu et al., 2017; Korpela et al., 2018; Wandro et al., 2018; Zwittink et al., 2018; Liu et al., 2019; Lu et al., 2020; Zwittink et al., 2020), whereas it was reduced after clarithromycin (macrolide) was utilized (Jia et al., 2020). There was a significant decrease in the relative abundance of the genus *Lactobacillus* spp. in preterm infants treated with clarithromycin, penicillin, and gentamicin (Barrett et al., 2013; Drell et al., 2014; Arboleya et al., 2015; Jia et al., 2020), but there was no change in a study that used cephalosporins (Hourigan et al., 2018).

### Antibiotic resistance due to antibiotic exposure

Even though antibiotics are used to treat pathogenic bacteria in preterm infants, their efficacy could be diminished by the presence of antibiotic-resistant bacteria. Three authors evaluated the effect of exposure to commonly used antibiotics on antibiotic resistance using shotgun metagenomic sequencing or quantitative PCR. The study by Gibson et al. (2016) evaluated resistance to 16 antibiotics and found multidrug-resistant members of the genera *Escherichia, Klebsiella*, and *Enterobacter*, the most notable observation being enrichment of a large number of overlapping antibiotic-resistant genes correlated with *Klebsiella pneumoniae* after exposure to ticarcillin–clavulanate and ampicillin. Using quantitative PCR, Ravi et al. found that the utilization of antibiotics, especially β-lactam, caused selection pressure to antibiotic-resistant bacteria in multiple patients with NEC (Ravi et al., 2017).

### Confounding

Dysbiosis is an unintended consequence of antibiotic therapy and is influenced by multiple factors, which can act in concert or separately. Therefore, assessment of confounding by detecting the presence of possible extraneous determinants is critical to obtaining valid results. In this review, preterm infant feeding was of interest, with milk (mothers’ own milk, donor milk, or formula) adjusted for in 16/21 studies. Mothers’ own milk modified the effect of antibiotic exposure in seven studies. The study by Gibson et al. (2016) observed that human milk significantly increased gut microbiota species richness, particularly *Bifidobacterium* spp., which had been significantly decreased by broad spectrum antibiotics, namely meropenem, cefotaxime, and ticarcillin–clavulanate (Gibson et al., 2016). Similarly, a study by Wandro et al. (2018) revealed an increase in the relative abundance of *Bifidobacterium* spp. after breastfeeding in ampicillin- and gentamicin-treated infants. Mothers’ own milk played a critical role in the restoration of pre-treatment microbiota in neonates exposed to antibiotics, independent of a “caesarean-section effect” (Korpela et al., 2018). Though not frequently reported as an assumed causal factor of interest, we found biological sex to confound the effect of antibiotics on gut microbiome. Infants exposed to antibiotics had significantly fewer *Proteus* spp. and significantly more *Citrobacter* spp. and *Clostridium perfringens* when these genera were present, with male infants having significantly more *Proteus* spp. than female infants. Neonatal stress due to parent–infant separation and pain from medical care procedures was associated with lower relative abundances of *Proteus* spp. and *Veillonella* spp. and a higher relative abundance of Gammaproteobacteria (D’Agata et al., 2019). In another study, an association was discovered between a lower abundance of *Veillonella* spp. and the neurotransmitter GABA, which has an impact on the gut–brain axis, with the potential for consequences in early-life development (Russell et al., 2021).

## Discussion

Antibiotic therapy has predictable intended effects (treatment) as well as unintended effects such as dysbiosis, which includes the loss of keystone microbiota, the loss of diversity, shifts in metabolic components, and a bloom of opportunistic pathogens (Vangay et al., 2015; Robertson et al., 2019). The microbiota disturbed by antibiotics contribute to human health through supplying vitamins and short-chain fatty acids, stimulating the immune system, and protecting against enteric infections (Nicholson et al., 2012; Spees et al., 2013). Such disruptions are very concerning in preterm infants because they are immature in almost every functional aspect, including motility, absorption, digestion, barrier function, and vascularization (Van Belkum et al., 2020). As a result, such perturbations will have an impact on immune, endocrine, metabolic, and other host developmental pathways (Robertson et al., 2019). In this review, we present evidence that has the potential to influence how antibiotics are prescribed to preterm infants. This is based on the belief that effective treatment is modern medicine’s stronghold, and patients and physicians alike expect diseases to be cured by appropriate interventions with unequivocal demonstration of minimal unintended consequences (Grobbee and Hoes, 2015).

Antibiotics are by far the most common drugs given to preterm infants in NICUs, despite surveys from large databases in the USA showing that the rate of culture-proven bacteremia in these infants at birth is only 1%–2% (Clark et al., 2006). Except where a prescription is indicated, the rationale to routinely and pre-emptively give these antibiotics shortly after birth is speculative rather than evidence based, and includes the possibility that preterm delivery may have been caused by infection in the mother, such as chorioamnionitis. (Cardetti et al., 2020). Moreover, compared with preterm infants who were not exposed to antibiotics, those exposed had high rates of NEC, sepsis, and death (Greenwood et al., 2014). In an effort to address this issue, we systematically selected studies that addressed the following concerns: (i) which taxa showed differential abundance as a result of antibiotic therapy in preterm babies?; (ii) how do the taxa present relate to each other?; and (iii) what are the consequences of dysbiosis? In the selection of the studies, the authors were mindful of dynamism of microbiota and thus adopted longitudinal data collection, which enabled investigation of trends and evolution of microbiota over time due to antibiotic treatment at the critical developmental stages of the preterm infants. Notably, antibiotic-induced microbiota alterations depended on the antibiotic spectrum, dose, and duration of treatment (Zimmermann and Curtis, 2019b).

The most commonly administered antibiotics for preterm infants in NICUs are amoxicillin, co-amoxiclav, benzylpenicillin, cephalosporins, gentamicin, vancomycin, clindamycin, and azithromycin (Centers for Disease Control, 2017). The typical postnatal antibiotic regimen consists of intravenously administered amoxicillin and/or gentamicin, and this combination was investigated in 10 studies, where it caused a decrease in *Bifidobacterium* spp. in nine studies, and complete disappearance in one of the studies (Wandro et al., 2018). These antibiotics are considered “microbiota friendly” because the depleted *Bifidobacterium* spp. is restored to pre-treatment levels within 30 days of stopping treatment (Jokela et al., 2022). A similar trend was observed in preterm infants exposed to vancomycin, a lipoglycopeptide, though the decrease in relative abundance of *Bifidobacterium* spp. caused an increase in *Enterococcus* spp. (Korpela et al., 2018). Some of the best antibiotics are semisynthetic macrolides such as clarithromycin and azithromycin, indicated for *Neisseria* and *Chlamydia* affecting newborn eyes (Patrinos et al., 2020). Two studies examining the impact of macrolides on the intestinal microbiota revealed a reduction in *Bifidobacterium* spp. A strong link has been identified between macrolide depletion of crucial microbiota and the development of asthma and obesity in childhood (Cox and Blaser, 2015). Clindamycin, a linconsamide, is a broad-spectrum drug used against aerobic Gram-positive cocci and anaerobic Gram-negative bacilli. Clindamycin should be used only in extreme cases to treat very serious infections because it virtually eliminates the intestinal microbiota and thus promotes colonization by *Clostridium difficile*, resulting in pseudomembranous colitis (Patrinos et al., 2020), and clindamycin-induced dysbiosis has been reported to last up to 2 years (Zaura et al., 2015). Cephalosporins such as cefazolin, ceftazidime, cefotaxime, cefuroxime, and cefoperazone were used in 12 studies. They are suitable replacements when target bacterial cells have developed penicillin resistance or when the patient is allergic to penicillin. Cephalosporins caused a relative increase in the abundance of *Enterobacteria*, mainly *E.coli*, and decreases in *Bifidobacterium* spp. and *Lactobacillus* spp. as well as significant decreases in species diversity. Additionally, cephalosporins were linked to antibiotic resistance genes (Gibson et al., 2016; Hourigan et al., 2018). This is consistent with other studies where the use of cephalosporins was linked to decreased levels of Enterobacteriaceae, primarily *E. coli*, and increased levels of Enterobacteriaceae other than *E. coli*, such as *Citrobacter* spp.*, Enterobacter* spp.*, Klebsiella* spp., and *Pseudomonas* spp. (Orrhage et al., 2000).

There was confounding by multiple factors including feeding practices, birth mode, sex, and stress, and these were taken into account in some but not all studies. Notably, breastfeeding was treated as a confounder appropriately, since breastmilk consists of core taxa of beneficial *Bifidobacterium*-rich microbiota, which are lacking in infants receiving donor milk, which is pasteurized and so does not include live microbes, or those receiving formula milk (Hunt et al., 2011). The live microbes in a baby’s own mother’s milk were beneficial through faster restoration of beneficial microbiota depleted by the antibiotics (Korpela et al., 2018). The contradictory findings on the phylum Bacteroidetes and *Bifidobacterium* spp. could be explained by different exposure to human milk oligosaccharides (HMOs), which play an important role in the development of the gut microbiota by nourishing specific microbiota (Davis et al., 2017). Furthermore, Bifidobacteria and Bacteroidetes are generally rare in premature infant feces unless supplemented with probiotics (Underwood et al., 2013). This is due to the aerobic environment, which favors aerotolerant taxa, primarily the Enterobacteriaceae family, in the neonatal gut. Within days, the aerotolerant taxa reduces oxygen levels and the intestinal lumen becomes anaerobic, allowing colonization by strict anaerobes dominated by *Bifidobacterium* spp., which is enhanced further by exclusive breastfeeding in the early days of life (Togo et al., 2019). In stressed male infants, D’Agata et al. discovered that *Proteus* spp. and *Veillonella* spp. were significantly reduced after antibiotic treatment (D’Agata et al., 2019). Russel et al. found a link between *Veillonella* spp. and the neurotransmitter GABA in a clinical trial that was part of this review, and that this link affected the gut–brain axis and early brain development (Clarke et al., 2013; Russell et al., 2021). Neonatal stress has a profound impact on the health and development of preterm infants, and physicians involved in their treatment and follow-up should be aware of this fact (Van Dokkum et al., 2021). The causes of neonatal stress include noise and bright lights, medical interventions and skin-breaking procedures, routine handling by physicians and nursing staff, and maternal separation (Mooney-leber and Brummelte, 2016). Neonatal stress has been linked to cognitive and motor development (Grunau et al., 2009), as well as the microbiota dysbiosis (D’Agata et al., 2019). This process is influenced by maternal genetics, as relative abundances of infant fecal *Veillonella* spp. are inversely correlated with concentrations of breastmilk-sialylated HMOs (Pace et al., 2021). The significance of birth route is well established. During vaginal birth, the infant is exposed to a variety of maternal microbes, including specific fecal microbes, which colonize the infant’s gut. This does not occur in infants born via C-section, who are colonized by microbiota from the environment, including potential pathogens from the hospital environment (Korpela, 2021). Antibiotics therefore have a greater effect on C-section babies, who might not receive human milk in the first few hours of life because the mother is unable to breastfeed due to the surgical procedures.

### Strengths and limitations

This systematic review’s strengths include an extensive literature search, clearly stated eligibility criteria, and a methodology that makes the review reproducible. Microbiome datasets are complex due to their high dimensionality and over-dispersion, making it challenging to directly test the association of microbiome composition with potential environmental factors using conventional statistical analysis tools (Xia et al., 2018). Nevertheless, most of the included studies utilized robust statistical models such as permutational multivariate analysis of variance (PERMANOVA), Mantel’s test, Dirichlet-multinomial model, and UniFrac distance model, and analyzed the microbiota data.

The main limitations of the studies were small sample sizes, a wide length of antibiotic therapy durations, and numerous antibiotics with different modes of action. The sample size influences power, and a power analysis should be performed during the sample collection period to estimate how many samples are required to provide sufficient power (e.g., 80%) to correctly identify a difference between groups. A few studies properly handled the potential confounding effects, such as mode of feeding, biological sex, mode of delivery, and neonatal stress. Additionally, different 16S rRNA regions were used to analyze stool samples and these were compounded by inconsistencies in the reporting of different alpha diversity metrics as well as taxonomic levels (phylum, class, family, order, genus, and species). The variations may be attributed to biological and technical differences resulting from different sequencing platforms, depths, or library sizes, which may account for differences between studies (Soneson and Delorenzi, 2013). Notably, all studies were conducted in Asia, Europe, and North America and none was conducted in low- and middle-income country (LMIC) settings, which have the highest proportion of preterm births.

## Conclusion

The review revealed that antibiotics cause a quantitative and qualitative dysbiosis depending on the spectrum and duration. After treatment discontinuation, the initial state was restored, but in some cases the microbiota restoration takes longer, creating a window of vulnerability for the host, because not all members of the microbial community are present to suppress pathogen blooms and thus prevent infection. The restoration to pre-treatment state was quickened by mothers’ own milk. Interestingly, there was a correlation between *Veillonella* spp. levels and GABA, a critical neurotransmitter for early brain development. These observations highlight the importance of restrictive and proper use of antibiotics in order to prevent dysbiosis, especially in situations where there is overuse of broad-spectrum antibiotics for conditions responsive to narrow-spectrum antibiotics (Rueda et al., 2019). There is a need for larger population-based studies with standardized reporting metrics in order to draw conclusions on the effects of exposure of antibiotics and their effects on microbiota. Moreover, it is important to focus studies on LMICs, where the burden of preterm infants is higher, and to determine if these are associated with future adverse health consequences.

## Data availability statement

This study analysed publicly available datasets. The datasets can be found in the articles that were used in the systematic review. The relevant data for this systematic review are summarised in **Supplementary Table 2**.

## Author contributions

MM, RN, and DW: conceptualized the study. MM and HK: designed the literature search. MM, SM, and HK: searched databases, screened records, extracted data, and assessed the risk of bias. MM: wrote the manuscript. MM, RN, and DW: revised the manuscript. All authors contributed to the article and approved the submitted version.

## Supplementary material

Supplementary file 1 Search terms for the research question: how does antibiotic therapy affect the gut microbiome in preterm infants when compared with no antibiotics?

Supplementary Table 2 Demographic, antibiotic, and taxonomic characteristics of the 21 selected studies. The data collected herein include author, year of publication, country, study design, study size (n), prescribed antibiotics, antibiotic-exposed group (n), controls (n), stool samples sequenced, follow-up time, length of treatment, 16S rRNA target, sequencing platform, α-diversity, and microbial taxa (phylum, family, genus, species). PT = preterm infants.

Supplementary Table 3 Newcastle-Ottawa Scale (NOS) for Assessing the Quality of Cohort Studies. This tool measures four domains including: participant selection, comparability, exposure, and outcome. The scoring is based on the number of stars, with longitudinal studies receiving up to nine stars. Based on the stars received, a study can be rated high, moderate, or low.
