# Supplementary material for: The impact of neonatal intensive care unit antibiotics on gut bacterial microbiota of preterm infants: a systematic review
Source: Front Microbiomes. 2023 Jul 28;2:1180565. doi: 10.3389/frmbi.2023.1180565 (PMC12993575; doi:10.3389/frmbi.2023.1180565)
Supplement: Supplementary file 1 — Search terms for the research question: how does antibiotic therapy affect the gut microbiome in preterm infants when compared with no antibiotics? [file Table_1.docx]

Supplementary Material

The impact of Neonatal Intensive Care Unit antibiotics on gut bacterial microbiota of preterm infants: A systematic review

**Martin M Mulinge ^1,2^*, Sylvia S Mwanza ^3^, Hellen M Kabahweza ^4^, Dalton C Wamalwa ^5^, Ruth W Nduati ^5^**

*** Correspondence:** Martin M Mulinge: [mmulinge@uonbi.ac.ke](mailto:mmulinge@uonbi.ac.ke)

**Supplementary file 1:** Search terms for the research question: How does antibiotic therapy affect the gut microbiome in preterm infants when compared to no antibiotics?

|  | **Pubmed** |
| --- | --- |
| 1 | "gut Microbiome" OR "gut Microbiota" OR dysbiosis OR "16S rRNA" AND infant OR neonate OR pediatric OR paediatric AND preterm OR "neonatal intensive care unit" OR NICU OR "very low birth weight" OR VLBW AND antibiotic* |
|  | **SCOPUS** |
| 2 | "gut Microbiome" OR "gut Microbiota" OR dysbiosis OR "16S rRNA" AND infant OR neonate OR pediatric OR paediatric AND preterm OR "neonatal intensive care unit" OR NICU OR "very low birth weight" OR VLBW AND antibiotic* |
|  | **EMBASE** |
| 3 | "gut Microbiome" OR "gut Microbiota" OR dysbiosis OR "16S rRNA" AND infant OR neonate OR pediatric OR paediatric AND preterm OR "neonatal intensive care unit" OR NICU:ti,ab,kw OR "Baby Friendly Initiative" OR BFI:ti,ab,kw OR "very low birth weight" OR VLBW OR VLBW:ti,ab,kw AND antibiotic* |
