# Supplementary material for: The impact of neonatal intensive care unit antibiotics on gut bacterial microbiota of preterm infants: a systematic review
Source: Front Microbiomes. 2023 Jul 28;2:1180565. doi: 10.3389/frmbi.2023.1180565 (PMC12993575; doi:10.3389/frmbi.2023.1180565)
Supplement: Supplementary Table 3 — Newcastle-Ottawa Scale (NOS) for Assessing the Quality of Cohort Studies. This tool measures four domains including: participant selection, comparability, exposure, and outcome. The scoring is based on the number of stars, with longitudinal studies receiving up to nine stars. Based on the stars received, a study can be rated high, moderate, or low. [file Table_3.docx]

Supplementary Material

The impact of Neonatal Intensive Care Unit antibiotics on gut bacterial microbiota of preterm infants: A systematic review

**Martin M Mulinge ^1,2^*, Sylvia S Mwanza ^3^, Hellen M Kabahweza ^4^, Dalton C Wamalwa ^5^, Ruth W Nduati ^5^**

*** Correspondence:** Martin M Mulinge: [mmulinge@uonbi.ac.ke](mailto:mmulinge@uonbi.ac.ke)

**Supplementary Table 3:** Newcastle-Ottawa Scale (NOS) for Assessing the Quality of Cohort Studies

| **#** | **Author** | **Selection** | **Comparability** | **Outcome** | **Rating** |
| --- | --- | --- | --- | --- | --- |
| 1 | Zhu et al., 2017, China | ★★★★ | ★★ | ★★★ | high |
| 2 | Zou et al., 2018, China | ★★★ | ★★ | ★★★ | moderate |
| 3 | Liu et al., 2019, China | ★★★★ | ★★ | ★★★ | high |
| 4 | Jia et al., 2020, China | ★★★★ | ★★ | ★★★ | high |
| 5 | Lu et al., 2020, China | ★★★ | ★★ | ★★★ | high |
| 6 | Drell et al., 2014, Estonia | ★★★★ | ★★ | ★★ | moderate |
| 7 | Barrett et al., 2013, Ireland | ★★ | ★★ | ★★ | low |
| 8 | Zwittink et al., 2018, Netherlands | ★★★ | ★★ | ★★ | moderate |
| 9 | d’Haens et al., 2019, Netherlands | ★★★ | ★ | ★★ | low |
| 10 | Zwittink et al., 2020, Netherlands | ★★★★ | ★★ | ★★★ | high |
| 11 | Arboleya et al., 2015, Spain | ★★★ | ★ | ★★ | moderate |
| 12 | Greenwood et al., 2014, USA | ★★★★ | ★★ | ★★ | moderate |
| 13 | Dardas et al., 2014, USA | ★★★ | ★★ | ★★★ | high |
| 14 | Gibson et al., 2016, USA | ★★★★ | ★★ | ★★ | moderate |
| 15 | Ravi et al., 2017, USA | ★★★★ | ★ | ★★ | moderate |
| 16 | Wandro et al., 2018, USA | ★★★ | ★★ | ★★★ | moderate |
| 17 | Hourigan et al., 2018, USA | ★★ | ★ | ★★ | low |
| 18 | D’Agata et al., 2019, USA | ★★★ | ★★ | ★★ | low |
| 19 | Lindberg et al., 2020, USA | ★★ | ★ | ★★ | low |
